# Increasing consultant-level staffing as a proportion of overall physician coverage improves emergency department length of stay targets

**DOI:** 10.1186/s12873-020-00399-8

**Published:** 2021-01-13

**Authors:** Dominic Jenkins, Sarah A. Thomas, Sameer A. Pathan, Stephen H. Thomas

**Affiliations:** 1 Department of Emergency Medicine, Hamad General Hospital, Doha, Qatar; 2 Imperial College London, London, UK; 3 School of Public Health and Preventive Medicine, Monash University, Melbourne, Australia; 4 Blizard Institute, Barts & The London School of Medicine, Queen Mary University of London, London, UK

**Keywords:** Emergency consultant, Emergency department, Length of stay, Less than four hours

## Abstract

**Objectives:**

One goal of Emergency Department (ED) operations is achieving an overall length of stay (LOS) that is less than four hours. The goal of the current study was to assess for association between increasing number of on-duty EM Consultants and LOS, while adjusting for overall (all-grade) on-duty emergency doctors’ numbers and other operational factors.

**Methods:**

This was a retrospective analysis of three years (2016–2019) of data, employing a unit of analysis of 3276 eight-hour ED shifts. The study was conducted using a prospectively populated ED database in a busy (annual census 420,000) Middle Eastern ED with staffing by Consultants and multiple non-Consultant grades (Specialists, fellows, and residents). Using logistic regression, the main predictor variable of “on-duty Consultant *n*” was assessed for association with the study’s primary (dichotomous) endpoint: whether a shift’s median LOS met the target of < 240 min. Linear regression was used to assess for association between on-duty Consultant *n* and the study’s secondary (continuous) endpoint: median LOS for the ED shift.

**Results:**

Multivariate logistic regression adjusting for a number of operations factors (including total EP on-duty complement) identified an association between increasing *n* of on-duty Consultants and the likelihood of a shift’s meeting the 4-h ED LOS target (OR 1.27, 95% CI 1.20 to 1.34, *p* < .0001). Multiple linear regression, which also adjusted for total on-duty EP *n* and other operational factors, also indicated LOS benefit from more on-duty Consultants: each additional on-duty Consultant was associated with a shift’s median LOS improving by 5.4 min (95% CI 4.3 to 6.5, *p* < .0001).

**Conclusions:**

At the study site, in models that adjusted for overall on-duty EP numbers as well as myriad other operational factors, increasing numbers of on-duty Consultants was associated with a statistically and operationally significant reduction in ED LOS.

**Supplementary Information:**

The online version contains supplementary material available at 10.1186/s12873-020-00399-8.

**Table:** 

***What is already known on this subject?*** Placemteent of senior doctors at ED triage, found significant LOS benefits from the practice. However, studies focusing on the effect of EM consultant in ED on the tMD, LOS, or adverse event rates present conflicting conclusions. The current study potentially adds to these reports in that we examined primary endpoint as shift median LOS meeting the 4-h target in a very high-volume ED. ***What this study adds***? Increasing numbers of on-duty EM Consultants was associated with a statistically and operationally significant reduction in ED LOS and improved odds of meeting 4-h A&E target.	

## Background

One of the major operations endpoints followed in the Emergency Department (ED) is length of stay (LOS, which as abbreviated in this report indicates ED LOS). The LOS is defined as elapsed time from patient presentation to the time of ED departure (whether for admission or discharge). The LOS is important because of its obvious relationship to efficiency and overcrowding, and also being linked to care quality and patient satisfaction [1–3]. Worldwide, LOS is perceived as important by both staff and patients [4–7]. In many EDs, the overall LOS target is four hours [2, 8].

The LOS has numerous components that have been subject to analysis. For example, the waiting time to see a physician (time to physician, tMD) should be minimized in order to expedite time-critical care, reduce overall LOS, and reduce rates of patients leaving without being seen (LWBS) [9, 10]. Assessments of LOS and its components (e.g. tMD, time from discharge order to ED departure) have stressed operational and medical benefits from throughput improvements [1, 11–13]. The presence of senior clinical-decision makers such as consultant grade doctors on the shopfloor are one of the factors considered to reduce LOS for patients presenting to ED. However, little is published on this topic.

The aim of this study was to assess whether a shift’s median LOS was influenced by increasing the proportion of on-duty EPs who were Consultant-grade. The goal was to adjust for a variety of operations factors, including patient census and total *n* of on-duty EPs, and ascertain whether changes in a shift’s proportional coverage by EM Consultants were associated with changes in the shift’s median LOS. The primary endpoint was defined as the dichotomous outcome: did the shift median LOS meet the target of four hours or less? The secondary endpoint was defined as the continuous outcome of median LOS.

## Methods

### Setting and physician staffing

The study was conducted at Hamad General Hospital (HGH), the only tertiary centre in the State of Qatar. During the study period, HGH ED was a 200-bed unit with annual census of approximately 420,000. The HGH institutional research ethics board approved the study, as a non-interventional assessment of operations data lacking patient identifiers.

The study ED is staffed in eight-hour shifts. During each shift, care is provided by 10–35 EPs of varying grades. Grades include EM residents (12/year in a four-year program, PGY1–4) and fellows (eight/year in a three-year program, who essentially function as would western-model PGY 5–7 residents), who complement the main workforce of EM Specialists and Consultants. The EP staffing makeup varies markedly from shift to shift, but over 99.5% of shifts’ EP coverage includes a Consultant *n* ranging from 1 to 12.

The total number of on-duty EPs during a given shift is tabulated by adding Consultant numbers to those of Specialists, fellows, and residents. The only exception to this rule of counting all EPs on duty is that 1st- and 2nd-year EM residents (PGY 1 and 2) are not included in the ED’s daily EP counts.

### Data source

The study ED uses an electronic medical record (EMR), Cerner Millennium FirstNet (Cerner, Kansas City, Missouri, USA). The EMR was deployed in May 2016. The EMR data are used daily to prospectively populate an ED operations database that does not contain patient identifiers or diagnosis information.

The ED database, maintained in Excel (Microsoft Corporation, Redmond, Washington USA) includes information on patient demographics and triage acuity (using the five-level Canadian Triage Acuity Scale or CTAS, in which Priority 1 is highest acuity). The database also tracks whether arrival mode was by ambulance (EMS). EP staffing (all grades) is tracked for each shift. Also tracked on a per-shift basis are ED operations “stress markers” of census, pending admissions (“boarders” who have been dispositioned for admission but who are awaiting movement from the ED), and rate of LWBS. The ED database does not contain information on work-up (e.g. lab tests ordered) or ED diagnosis.

The ED database uses the EMR-calculated LOS, without any “cleaning” of the data. The EMR starts the LOS time interval with patient arrival and registration (either at an ED desk for lower-acuity cases, or at bedside for higher-acuity cases). After patient’s complete evaluation, the nurse providing care records a departure time when the patient actually leaves the ED (regardless of destination). The LOS is thus the time interval between registration and ED departure.

### Time frame and units of analysis

The data covered a three-year period. The study time frame was defined to start six weeks after the study ED moved from paper charting to EMR; the six-week interval was designed to allow for EMR run-in time. A study time frame of three 52-week years was constituted, to generate an overall study period of July 2016 through June 2019.

The unit of analysis was the ED shift. All EPs work either early (0700–1500), late (1500–2300), or night (2300–0700) shifts. With three shifts per day over three years, the total number of shifts was thus 3276. This defined the study set for the primary and secondary endpoints.

### Analysis

Analysis and graphs were executed using Stata 16MP (StataCorp, College Station, Texas, USA). Statistical significance was defined as *p* < .05, and confidence intervals (CIs) are reported at the 95% level.

Categorical variables are reported as proportions with binomial exact CIs. Continuous variables (which were found non-normal by quantile plotting and Shapiro-Francia testing) are reported as median with interquartile range (IQR). Univariate analyses, executed to screen covariates for exploration in multivariate modelling, were executed using simple regression.

For the analysis of the primary dichotomous endpoint (“shift median LOS <240 minutes”), logistic regression was utilized using methods described in detail by Hosmer and Lemeshow [14]. Covariates with univariate *p* < .20 were evaluated for inclusion in modelling. Post-estimation model evaluation included assessments of calibration (Hosmer-Lemeshow goodness-of-fit), discrimination (*C* statistic), and specification (link function test).

For the analysis of the secondary continuous endpoint (shift median LOS), linear regression was utilized with modelling approach similar to that as described for the primary endpoint. Post-estimation evaluation included calculation of adjusted *R*^2^ and (if heteroskedasticity was suggested by residual-vs.-fitted plotting) robust CIs. Model assumption violations regarding the main predictor variable were assessed by plotting residuals vs. levels of the Consultant *n*. Sensitivity to outliers was assessed by recalculating whether the estimated Consultant *n* effect on median LOS was changed by removing extreme outliers.

For both primary (logistic) and secondary (linear) regression models, marginal probabilities were calculated using Stata. Regression-predicted effects of Consultant staffing on LOS were plotted using marginal predictive probability plotting [15].

### Patient and public involvement

Patients or the public WERE NOT involved in the design, or conduct, or reporting, or dissemination plans of our research. However, the study aim, and objectives are very much aligned to the best of public interest and priorities of public health set to meet national health strategy.

## Results

### Overall operations and demographic characteristics for *n* = 3276 shifts

During the three-year study period, the ED census was 1,260,579. These patients were seen over 3276 eight-hour shifts. Information on ED operations and patient parameters for the study shifts is shown in Table 1

**Table 1:** Characteristics of 3276 Emergency Department (ED) shifts

Variable	Median	Range, interquartile range
***Operations markers***		
Patient census	385	193–705, 293–459
*n* of pending admissions at shift start	47	0–110, 35–59
Proportion left without being seen	0.109	0.006–0.304, 0.078–0.141
Median length of stay (minutes)	236	84–1504, 204–272
***Patient demographics and acuity***		
Proportion of females	0.310	0.164–0.444, 0.270–0.342
Proportion of Qatari nationals	0.2	0.076–0.374, 0.158–0.238
Proportion of pediatric (age < 18) cases	0.087	0.010–0.211, 0.056–0.129
Proportion of geriatric (age > 64) cases	0.040	0.004–0.083, 0.032–0.048
Proportion of lowest-acuity triage scores^a^	46.4	12.3–79.0, 41.4–56.2
***Total number of on-duty physicians***		
All grades combined	25	10–35, 23–27
Consultants	6	0–15, 4–7
Specialists	11	0–22, 9–14
Fellows	6	0–19, 4–8
Residents	1	0–16, 0–3

^a^Triage scores 4 or 5 on a five-point scale

.

### Length of stay results

**Fig. 1:**
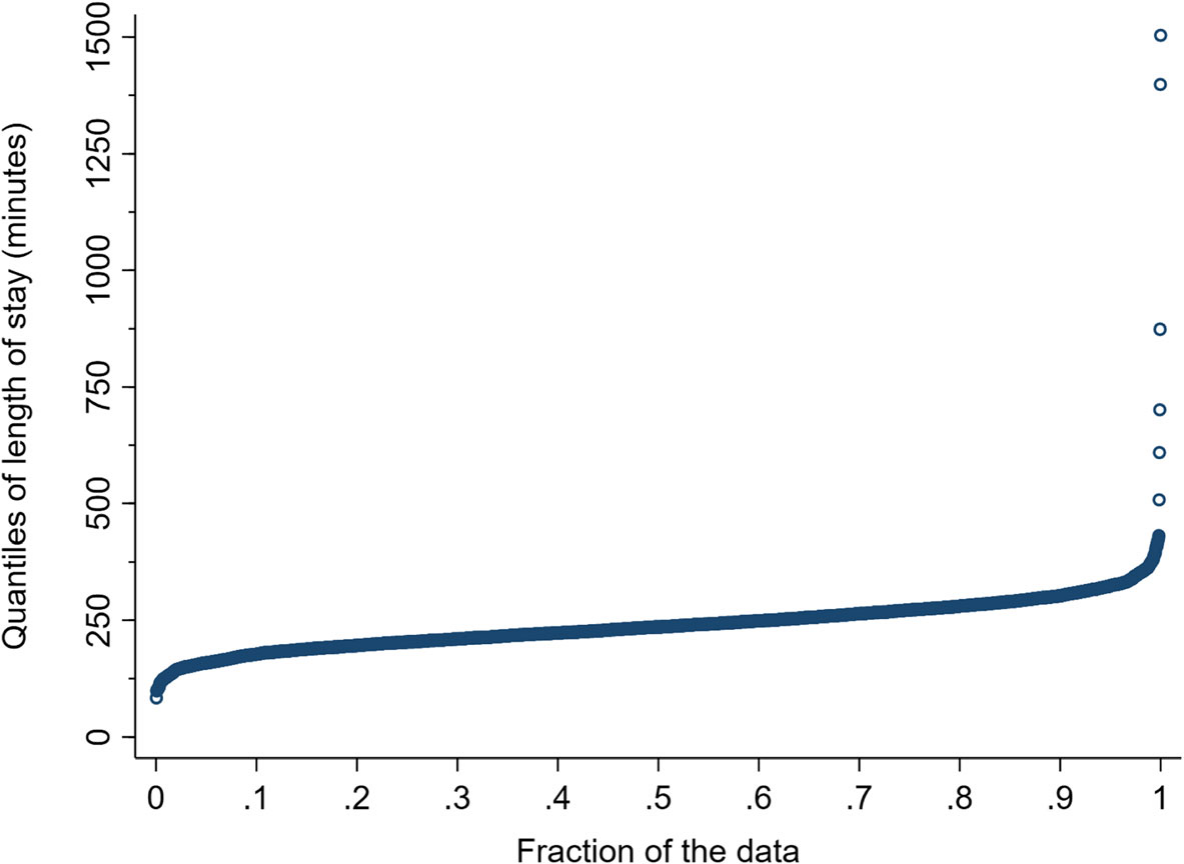
Quantile plot of 3276 shifts’ median length of stay (LOS)

As shown in Table 1, the median LOS over the 3276 shifts was just under the 240-min target. The LOS target of four hours or less was met in 1748 (53.4%) of 3276 shifts. Figure 1 shows the quantile plot of the 3276 shifts’ median LOS. In particular, the plot demonstrates the positive (right) skew of the data (note spike at right side of plot).

### Primary endpoint (likelihood of meeting shift length-of-stay target): logistic regression results

Each of the operations and demographic variables was then assessed for univariate association with the shift’s median LOS falling within the four-hour target. Details of univariate analyses and model-building for the logistic regression model are provided in Additional file 1. The primary independent variable (Consultant *n*) and five covariates were identified as having a significant adjusted association with the likelihood of a shift median’s LOS being less than four hours.

The final multivariate logistic regression model (as outlined in Additional file 1), containing the primary independent (predictor) variable – on-duty Consultant *n* – and five covariates was generated and outlined in Table 2

**Table 2:** Logistic regression model: Factors associated with likelihood of shift median length of stay (LOS) falling within four hours

Variable	Odds ratio (95% confidence interval)	***p***
On-duty Consultant *n*	1.27 (1.20–1.34)	<.0001
Total on-duty physicians	0.84 (0.81–0.88)	<.0001
Study month	0.95 (0.94–0.95)	<.0001
Shift time (day, evening, night)	1.67 (1.36–2.06)	<.0001
Shift census (per 100 patients)	0.73 (0.63–0.85)	.0001
Proportion left-without-being-seen cases on shift	0.73 (0.71–0.75)	<.0001

.

**Fig. 2:**
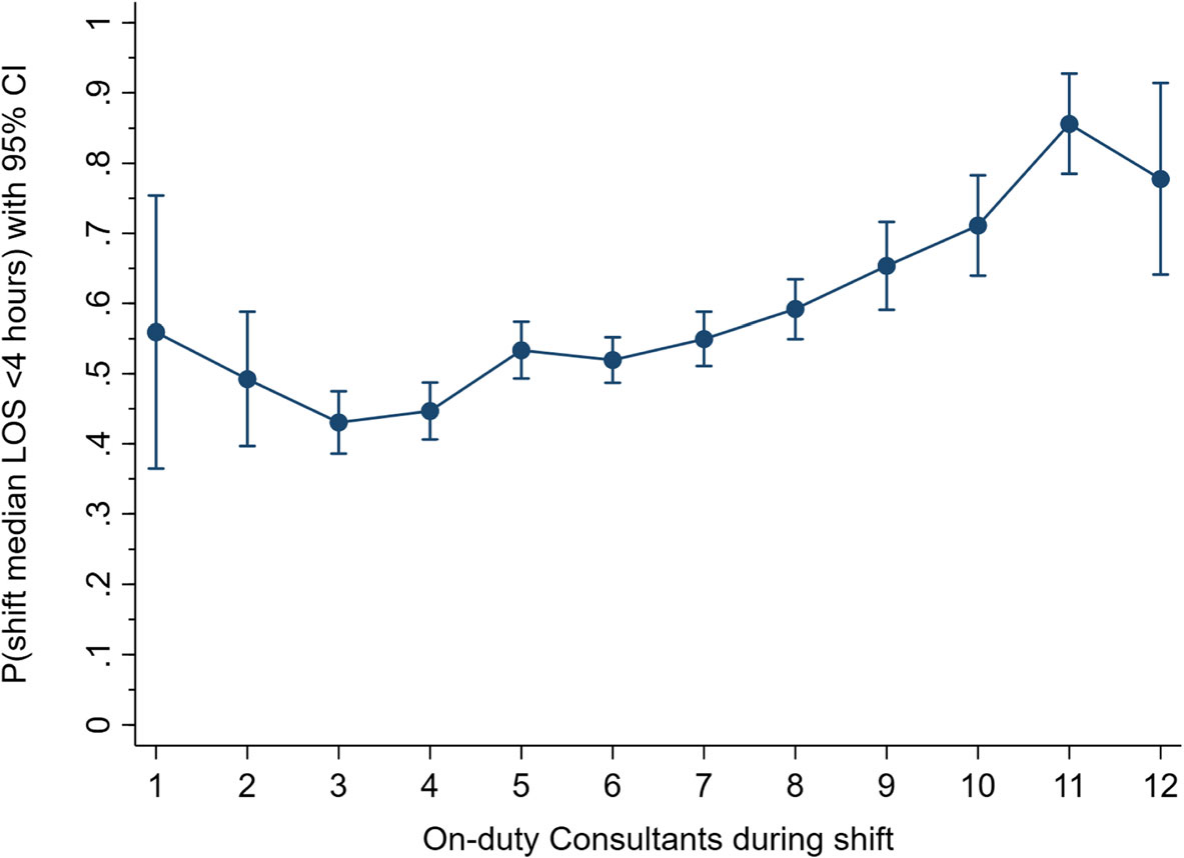
Consultant on-duty *n* and adjusted likelihood (with 95% confidence interval, CI) of meeting shift length of stay (LOS) target

Since nearly all (> 99.5%) ED shifts are staffed by a Consultant *n* within the range of 1 to 12, marginal probability plotting was used to adjust for the model’s covariates and illustrate the Consultant *n* effect on a shift’s likelihood of meeting the LOS target as shown in Fig. 2.

### Secondary endpoint (shift median length of stay): linear regression results

The effect of Consultant *n* on median LOS was assessed using the same endpoints that had been assessed for association with a shift’s likelihood of meeting the LOS target (Additional file 1). The primary independent variable (Consultant *n*) and eight covariates were identified as having a significant adjusted effect on the shift median LOS. Three were calendar covariates (Friday/non-Friday status, shift time, and study month), three were operations indicators (census, total on-duty EPs, and LWBS proportion), and two were related to patient types seen (shift proportion of Qatari nationals, shift proportion of low-acuity cases).

**Fig. 3:**
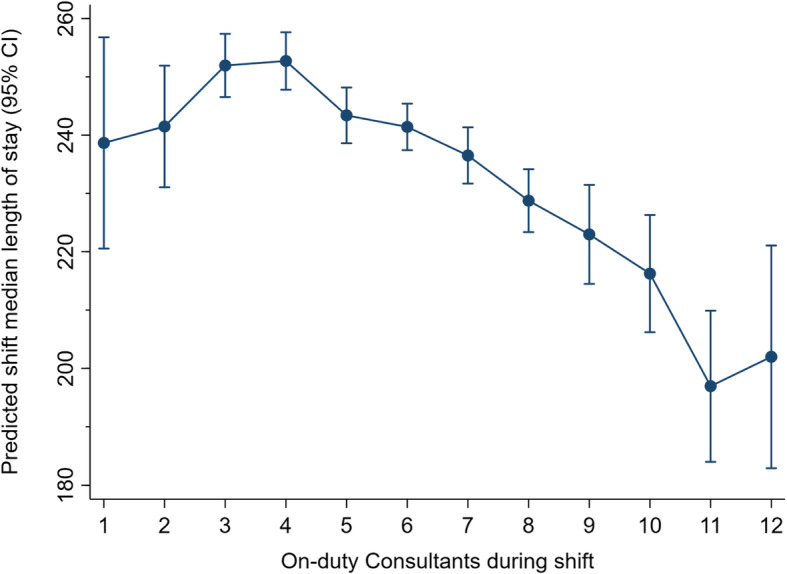
Consultant on-duty *n* and adjusted median length of stay (with 95% confidence interval, CI)

In multivariate modelling (as outlined in Additional file 1), a final model containing the primary independent (predictor) variable – on-duty Consultant *n* – and eight covariates was generated as outlined in Table 3. Since nearly all (> 99.5%) ED shifts are staffed by a Consultant *n* within the range of 1 to 12, marginal probability plotting was used to adjust for the model’s covariates and illustrate the Consultant *n* effect on a shift’s median LOS. The plot is shown in Fig. 3.

**Table 3:** Linear regression model: Factors associated with likelihood of shift median length of stay (LOS) falling within four hours

Variable	Odds ratio (95% confidence interval)	***p***
On-duty Consultant *n*	−5.44 (−6.53 to − 4.35)	<.0001
Total on-duty physicians	2.55 (1.76–3.34)	<.0001
Study month	1.60 (1.39–1.81)	<.0001
Shift time (day, evening, night)	−24.2 (− 29.6 to − 18.8)	<.0001
Shift occurrence on a Friday	−11.5 (.16.9 to −6.13)	<.0001
Shift census (per 100 patients)	3.44 (0.14–6.74)	.036
Proportion left-without-being-seen cases on shift	6.43 (6.00–6.89)	<.0001
Proportion of Qatari nationals on shift	−2.25 (− 2.89 to −1.61)	<.0001
Proportion of low-acuity cases on shift	−2.52 (− 2.88 to − 2.16)	<.0001

## Discussion

LOS is followed in virtually every ED. Streamlining care and minimizing LOS has overt benefits, such as optimizing use of ED beds; reduced LOS also has the potential to reap other (perhaps less intuitive) benefits: medical care quality, patient satisfaction, and LWBS rates [1, 16]. A 2019 report focusing on factors driving ED patient satisfaction found that LOS and its tMD component were two of the most critical determinants, with both being more important than EP-related characteristics [7].

As exhibited by the models reported in this study, there are multiple factors that influence LOS broadly classified as consultant staffing, calendar and time-of-day factors, and operations factors. Without diminishing the importance of these many factors that affect LOS, this study focused on the LOS effects seen with changes in on-duty Consultant *n*. Other variables were included not for investigational reasons, but rather to allow the Consultant-staffing analysis to adjust for as many important covariates as possible. The findings regarding non-staffing covariates in the final estimated models for both the primary and secondary endpoints with possible explanations are detailed in Additional file 1.

### Findings relating to physician staffing and length of stay

The main current study findings are best summarized as follows: Even when adjusting for a variety of factors including total on-duty EP *n*, increasing the number of on-duty EM Consultants was associated with improved LOS performance. These findings were consistent regardless of whether a shift’s median LOS was assessed as a dichotomous outcome (“met the LOS goal”) or as a continuous variable. Within the usual ranges of per-shift staffing at the study site for Consultants [1–12] and total EPs (10–35), each additional Consultant was associated with a 5.4-min reduction in shift median LOS and a 27% increase in odds of that shift’s LOS being four hours or less.

In ED operations studies from Europe, Australia, Canada, and the USA, LOS reductions have been labelled significant when they have been in the range of 8–11 min [17–20]. These studies have often entailed combined assessment of substantial system changes including addition of multiple providers (both Consultants and nurse practitioners). We conclude that a per-Consultant LOS improvement of 5.4 min is operationally important. The relevance of the four-hour threshold is more subjective, but in our system, there is (substantial) administrative pressure to have the proportion of “met-goal” shifts as high as possible.

Others have examined the effects of staffing the ED with higher-grade EPs. A Portuguese study from 2017 demonstrated that as EDs move from historical staffing patterns of non-EM staffing towards dedicated EPs, there are improvements in both costs and LOS [21]. A 2016 systematic review of studies assessing placement of senior doctors at ED triage, found significant LOS benefits from the practice [22]. More recently, a 2019 study from the UK found that moving towards Consultant presence on overnight shifts was not associated with improved tMD, LOS, or adverse event rates [23]. The authors of that study noted that their findings were contradictory to data reported previously; two other sets of investigators had found that Consultant presence in the ED was associated with improved LOS as well as other operational and clinical benefits [24, 25]. Overall, the data from EDs that (like our centre) are “high-volume” seems to indicate benefit from increased Consultant presence. A UK ED with annual census 100,000 found that moving towards a Consultant presence during overnight shifts resulted in a 21-min reduction in LOS [24]. Another study from a busy London ED found that Consultants’ involvement reduced the extent of patient workup and had salutary effect on LOS [8].

It is important to emphasize that, among other covariates, the current analysis adjusted for total numbers of EPs. While adding a Consultant to the triage (“out-front”) area has had some reports of success [18], simply increasing overall EP staffing has not always had statistically significant influence on LOS [26]. Other study strengths included the size of the studied set of shifts, the capability to adjust for multiple covariates, and the fact that the two models looking at different LOS facets produced consistently favourable results. Before any conclusions can be drawn from our results, however, a number of study limitations must be considered.

### Limitations

The study’s results are certainly not definitive, and our findings should be considered only as potentially adding information to the existing evidence base. The analysis suffers from both internal and external validity problems.

First, the study was performed on data that were EMR-reported and thus objective, but not necessarily accurate. No data were discarded, despite the presence of outliers (e.g. the shift median LOS exceeding 25 h) that were almost certainly incorrect. Rather than make difficult and inherently subjective decisions about which data were “probably wrong,” we chose to analyse the data just as reported in the database. Some steps were taken to mitigate the risks posed by faulty data. First, the use of central tendency (shift median LOS) was intended to blunt the effect of outliers inherent in the study centre’s operational database. The dichotomous endpoint of shift median LOS < 4 h was designed to be particularly insensitive to outlier data; this is the primary reason for selection of the dichotomous measure as the main study endpoint.

Related to the issue of errors in the database were missing data. Of the 1,260,579 cases seen during the study period, LOS was reported by 1,117,406 (88.6%). Advance knowledge of the missing-data problem contributed to the a priori decision to mitigate methodological risk by using shift as the unit of analysis; all shifts had a median LOS. Since the aim of the study was to assess EP staffing, not model LOS, the decision was made to simply analyse the data “as-is” rather than attempt to “correct the data” with techniques such as multiple imputation (such techniques are potentially useful but come with their own drawbacks). The missing-data problem, while not easily quantified, remains a major study limitation.

An additional study limitation was the fact that the ED database does not track certain information. The current study adjusted for many of the commonly discussed non-staffing factors that are likely to affect LOS. Time-related factors (such as day of the week), shift census, and proportions of various age groups are known LOS influencers [27]. The current study assessed time factors and other variables, such as triage acuity [28], with known LOS association, but many important data points affecting LOS were not analysed. Diagnosis and related information on work-up (including lab or radiology testing and consultation with other services), are not included in the ED operations database, but these variables are known to affect LOS [3, 29]. Failure to adjust for these factors, either as influencers of LOS or as putative mechanisms for Consultant staffing-related LOS improvements, is an important limitation of this study.

A final limitation deals with external validity. The study site is populated by Consultants and Specialists primarily, with additional staffing assistance from fellows and (to a lesser degree) residents. Other EDs with different staffing – different by number, grade, or even quality – may not experience the same benefits from Consultant presence in the ED.

## Conclusion

In modelling that adjusted for a number of important operational factors, incremental changes of Consultant staffing were associated with significant improvements in LOS at the study ED. The study’s results are certainly not definitive, and our findings should be considered only as potentially adding information to the existing evidence base.

## Supplementary Information

**Additional file 1.**

## Data Availability

The datasets generated and/or analysed during the current study are not publicly available due to anonymity but are available from the corresponding author on reasonable request.

## Abbreviations

ED: Emergency Department
EMR: Electronic Medical Record
HGH: Hamad General Hospital
LOS: Length of Stay
LWBS: Leaving Without Being Seen
