# Supplementary material for: Increasing consultant-level staffing as a proportion of overall physician coverage improves emergency department length of stay targets
Source: BMC Emerg Med. 2021 Jan 13;21:5. doi: 10.1186/s12873-020-00399-8 (PMC7805094; doi:10.1186/s12873-020-00399-8)
Supplement: Supplementary file 1 — Additional file 1. [file 12873_2020_399_MOESM1_ESM.docx]

**Appendix 1. Logistic regression model-building for endpoint “shift median length of stay under 4 hours”**

This appendix provides information regarding specifics of generating and evaluating the model for the study’s primary dichotomous endpoint: whether or not a given shift’s median LOS fell within 4 hours. The appendix outlines the steps entailed in building and assessing suitability of the model reported in the manuscript.

The initial step was execution of univariate logistic regression of the following covariates, to see which met the threshold of *p* < .20, for inclusion in multivariate regression of LOS on Consultant on-duty *N*. The following covariates were examined (those marked with * had *p* values below the threshold and were included in the multivariate modeling):

- **Total on-duty physicians* (total *n*, all grades of on-duty physicians)
- **Friday* (whether the shift occurred on a Friday)
- **Month* (on which month during the study’s 36 months)
- **Ramadan* (did the shift occur during the month of Ramadan)
- **Shift time* (shift time: early 0700-1500, late 1500-2300, or overnight 2300-0700)
- **Census* (total patient registrations during the shift)
- *Shift proportion of females*
- *Shift proportion of Qatari nationals*
- *Shift proportion of ambulance cases*
- *Shift proportion of low-acuity cases*
- *Shift proportion of pediatric cases* (age<18 defined pediatric cases)
- **Shift proportion of geriatric cases* (age>64 defined geriatric cases)
- **Shift proportion of left-without-being-seen cases*
- *Number of pending-admit cases (“boarders”) at shift commencement*

The above covariates marked with * were included in a logistic regression model, and the model output contained three non-significant covariates: *Ramadan* (*p* = .732), *Friday* (*p* = .392), and *shift proportion of geriatric cases* (*p* = .092). All other covariates in the model were significant (by Wald testing) at *p* < .001.

The model was re-run after removing the *Ramadan* covariate, and the resulting model yielded two non-significant covariates: *Friday* (*p* = .371), and *shift proportion of geriatric cases* (*p* = 091). All other covariates in the model were significant (by Wald testing) at *p* < .001.

The model was re-run after removing the *Friday* covariate, and the resulting model yielded one non-significant covariate: *shift proportion of geriatric cases* (*p* = .103). All other covariates in the model were significant (by Wald testing) at *p* < .001.

The model was re-run after removing the *shift proportion of geriatric cases* and the resulting model had all *p* values <.001 by Wald testing. That model was the final model reported for the dichotomous outcome logistic regression testing. To calculate final reported *p* values, the likelihood ratio test *p* was calculated for each of the variables that was included in the final model. These are the *p* values reported in the manuscript table.

The model was demonstrated to have good calibration (Hosmer-Lemeshow goodness-of-fit *p* = .695) and discrimination (C statistic or AUC .82 as shown in Figure Appendix1). Link testing identified no evidence for omitted variables or incorrect link-function specification (hat-squared *p* = .098).

***Figure Appendix1.*** Area under curve (*C* statistic) for logistic regression model


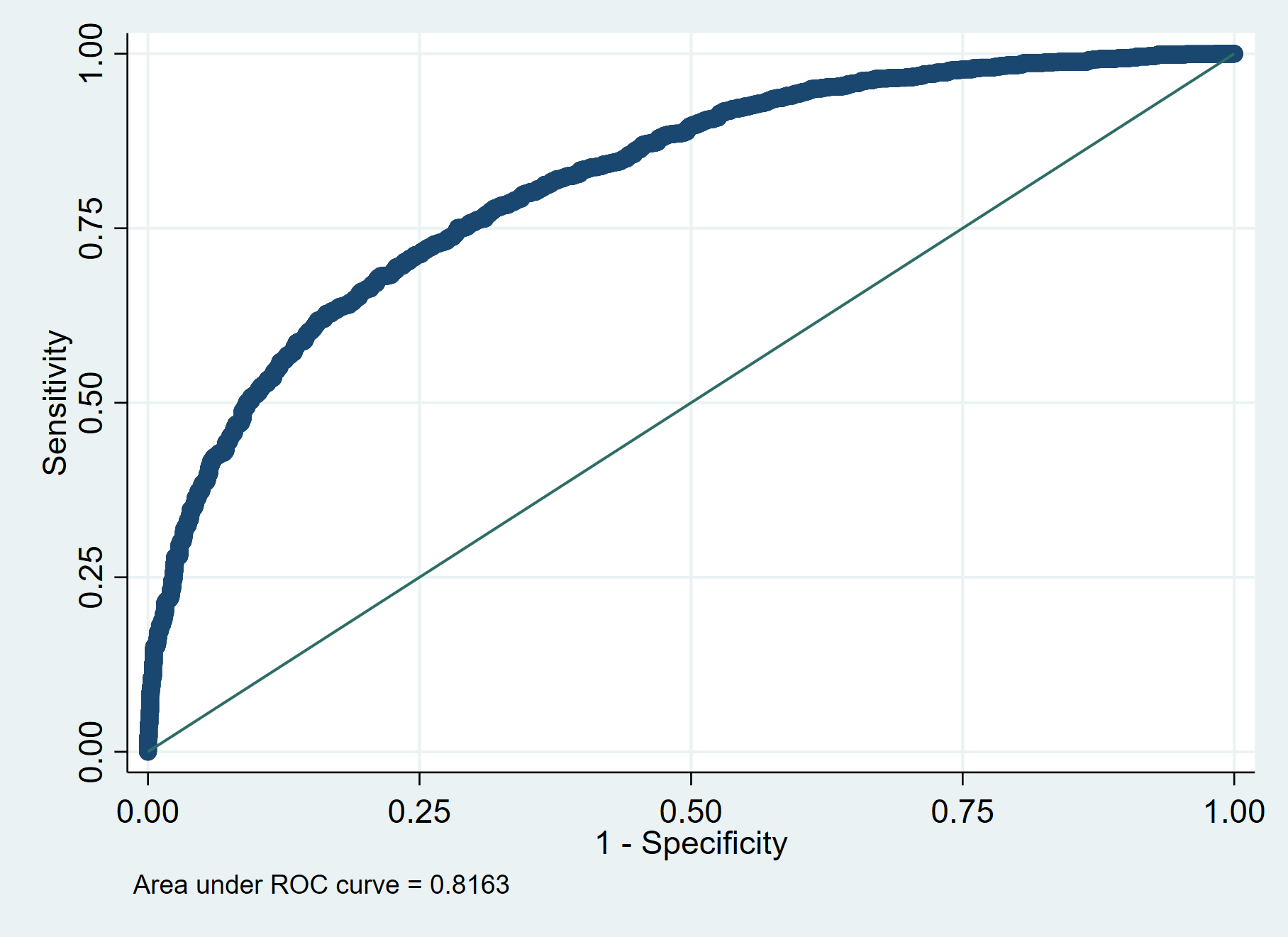


**Appendix 2. Linear regression model-building for endpoint “shift median length of stay”**

This appendix provides information regarding specifics of generating and evaluating the model for the study’s secondary continuous endpoint: shift median LOS. The appendix outlines the steps entailed in building and assessing suitability of the model reported in the manuscript.

The initial step entailed assessing the same univariates that had been assessed for logistic regression (as outlined in Appendix 1). The covariates that met the *p* <.20 threshold for exploration in multivariate modeling included: total patients and physicians, shift time of day (morning, evening, or overnight), whether shift occurred on a Friday or during Ramadan, study month, and shift proportions of Qataris, low-acuity cases, pediatrics, and LWBS. The *p* values for each of the preceding were <.001 with the exceptions of proportions of Qatari (*p* = .027) and pediatrics (*p* = .049). Covariates that did not meet the threshold for modeling inclusion were: proportion of female (*p* = .436) and geriatric (*p* = .576) cases, and number of pending admissions (*p* = .575).

The above covariates that met criteria for inclusion in modeling were included in a linear regression model, and the model output contained two non-significant covariates: *Ramadan* (*p* = .643), and *shift proportion of pediatric cases* (*p* = .468). All other covariates in the model were significant (by Wald testing) at *p* < .001.

The model was re-run after removing the *Ramadan* covariate, and the resulting model yielded one non-significant covariate: *shift proportion of pediatric cases* (*p* = .443). With the exception of shift census (*p* = .030), all other covariates in the model were significant (by Wald testing) at *p* < .001.

The model was re-run after removing the *shift proportion of pediatric cases* covariate, and the resulting model yielded no non-significant covariates. The *shift census* covariate *p* was .041 and all other covariates were significant (by Wald testing) at *p* <.001.

To calculate final reported *p* values, the likelihood ratio test *p* was calculated for each of the variables that was included in the final model. These are the *p* values reported in the manuscript table.

The residuals-*vs.*-fitted plot (shown below in Figure Appendix2a) did not clearly indicate heteroskedasticity. However, some outliers were suggested, so robust CIs were calculated for the main predictor variable; these are the CIs that are reported in the manuscript.

***Figure Appendix2a.*** Residual *vs.* fitted plot for linear regression model


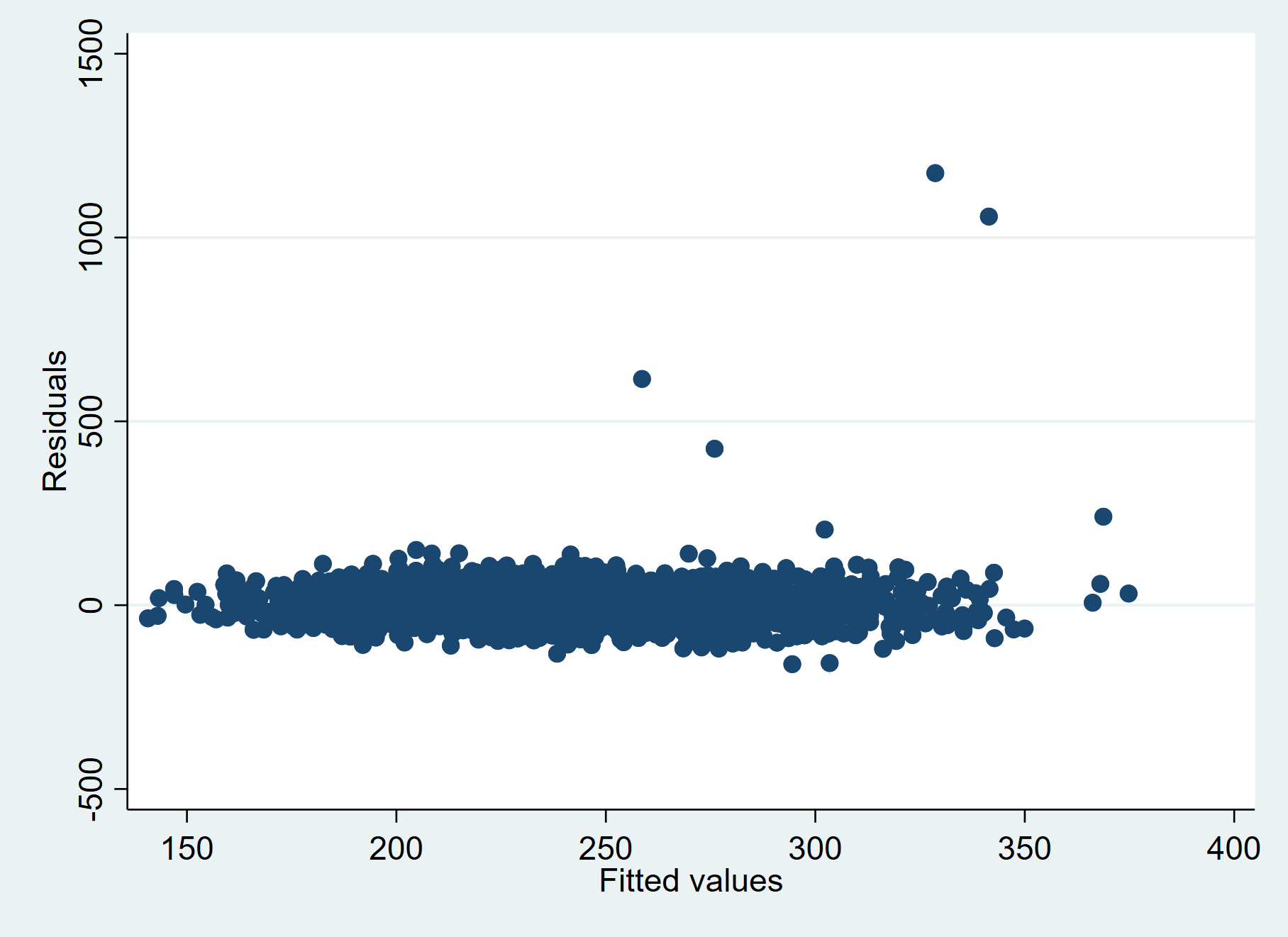


To assess for signs of violation of regression assumptions by the main predictor variable of interest, residuals were plotted against predicted values for Consultant *n*. In this plot (Stata’s *rvpplot* procedure), there were no obvious patterns suggesting violation of assumptions (Figure Appendix2b).

The median shift LOS (a continuous variable, and thus one containing more information) was known to be more subject to outliers. While no LOS data were discarded for any findings presented in the results section, Appendix 2 outlines sensitivity analysis demonstrating that discarding the five largest outliers had no appreciable effect on the estimated 5-minute LOS reduction per Consultant. There is little reason to think that LOS findings would be biased one way or another by changing proportional Consultant coverage, but despite risk-mitigation measures taken for both logistic and linear regression, inevitable errors in the study database remain a shortcoming of the current analysis.

This study’s use of a four-hour goal for LOS is not unusual.[^2^](#_ENREF_2)^,^[^8^](#_ENREF_8) The ultimate aim for the study ED would be to have the goal met for all patients (or at least met by far more than half). However, this study’s identified median LOS and proportion of cases meeting the four-hour goal are not inconsistent with LOS data from other EDs.^[21](#_ENREF_21)^

We chose to not execute log-transformation (or some other Box-Cox transformation) of the LOS data, but instead to assess median LOS by shift. Our LOS data were positively skewed to an extent that methodologists have suggested renders LOS modeling quite difficult, even with log-transformations and advanced techniques such as generalized linear modeling with various link functions.[^22^](#_ENREF_22)^,^[^23^](#_ENREF_23) We emphasize that the purpose of the current analysis was not to provide a full prediction of a shift’s median LOS – at which predictive task the current study fails, due to accounting for only 34% of the LOS variation. We instead set out to achieve a goal, unrelated to the model’s *R*^2^ value, of calculating an unbiased estimate of the LOS effects associated with changing *n* of on-duty Consultants. In fact, the 0.34 *R*^2^ from this study’s linear regression was higher than many reported corresponding calculations from large-scale LOS databases; similar methodologies from national-level intensive care unit studies in Australia and the Netherlands used myriad modeling efforts and only achieved *R*^2^ values ranging up to 0.22.[^22^](#_ENREF_22)^,^[^23^](#_ENREF_23)

***Figure Appendix2b.*** Residual *vs.* predicted plot for Consultant *n* variable


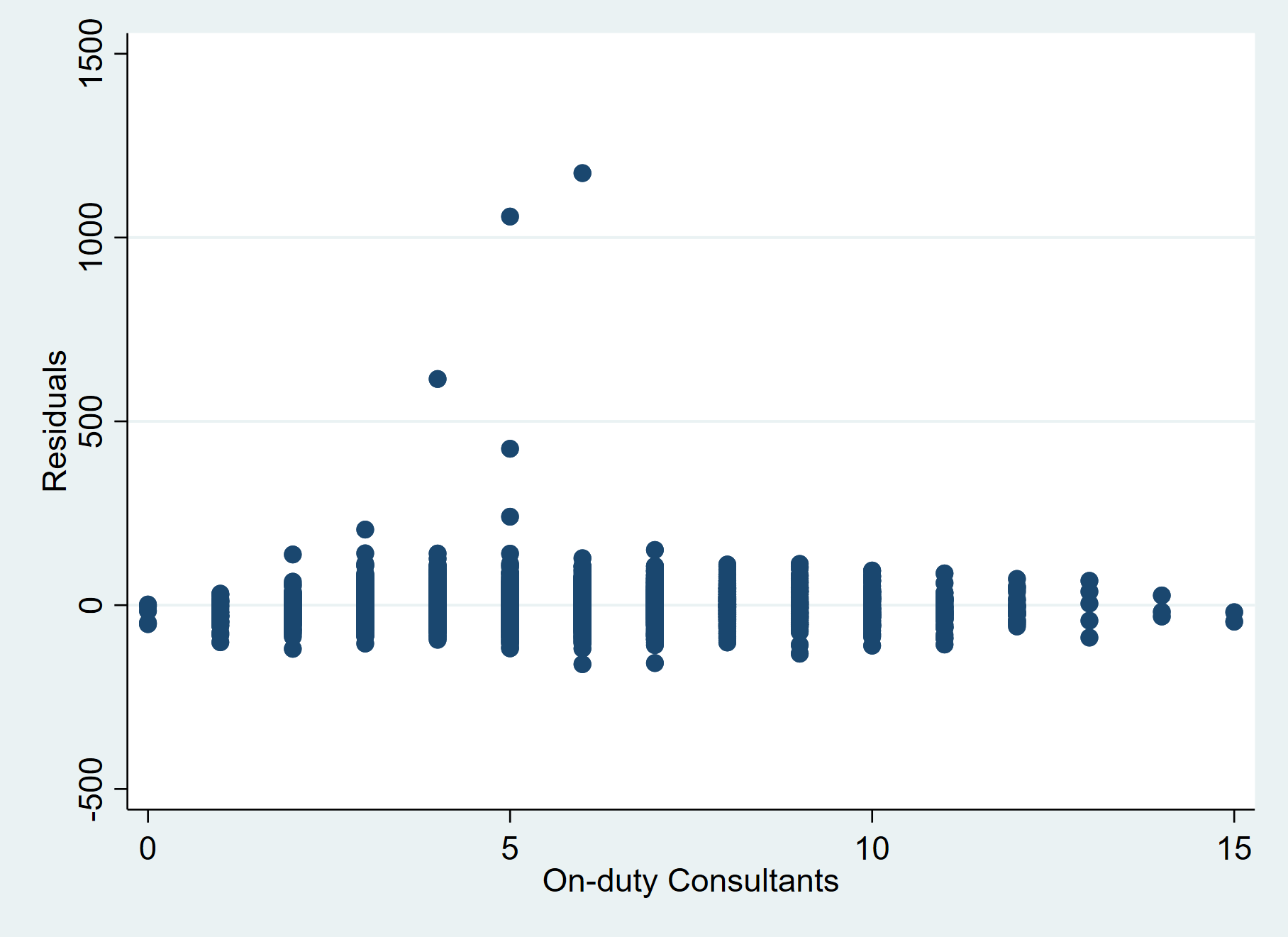


The final assessment of the linear regression model was the re-running of the model after excluding the four outlier shifts (shift numbers 469, 568, 570, and 877) that were identified on each of the above two plots. These four outliers were confirmed as such, by plotting leverage *vs.* residual^2^ and labeling the outlier shifts (see Figure Appendix2c below).

***Figure Appendix2c.*** Leverage *vs.* residual-squared plot for outlier identification


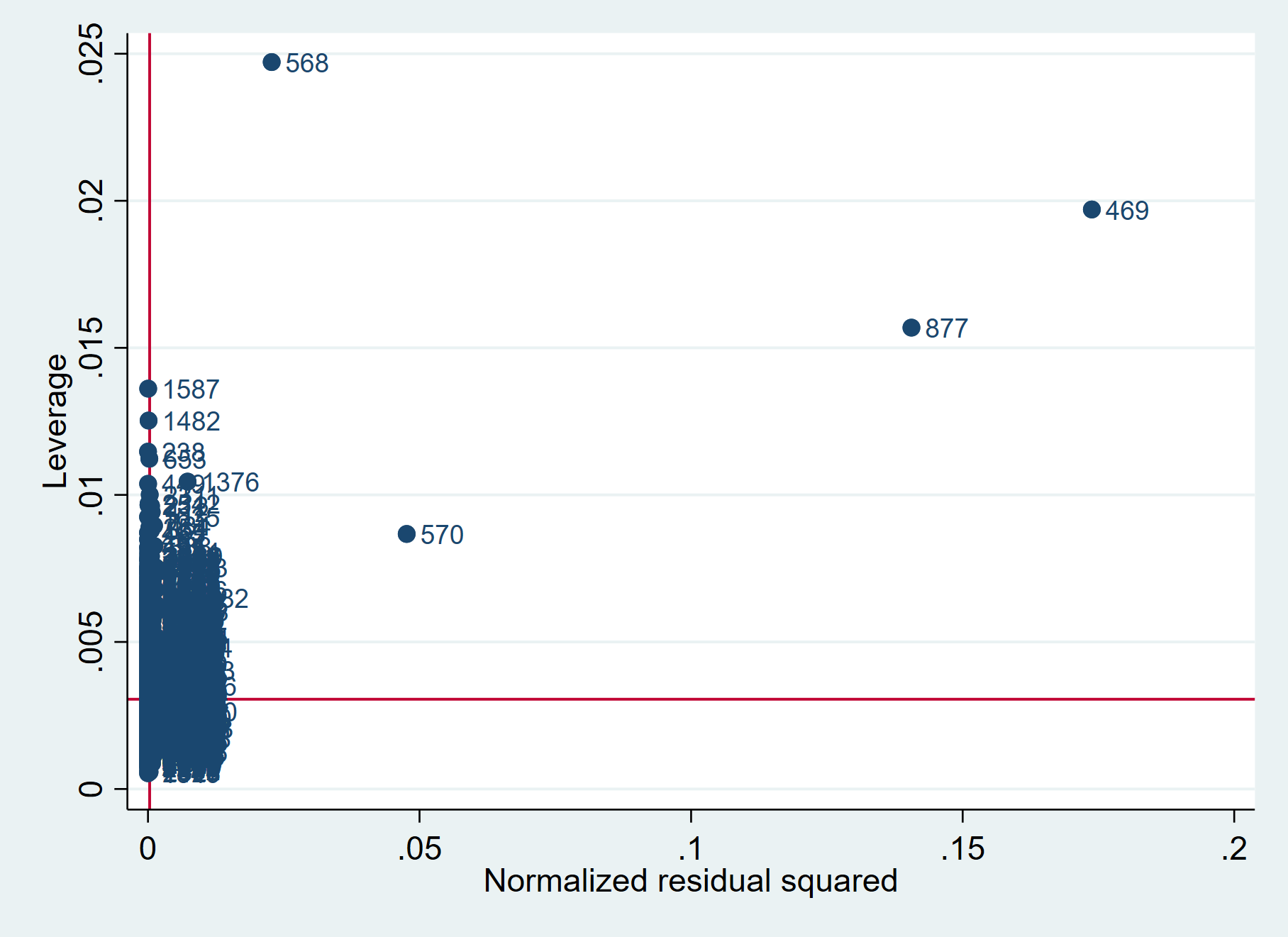


Re-execution of the linear regression model with exclusion of those four shifts did not result in any appreciable change from the main model (as reported in the manuscript) calculated effect estimate (5.0), CI (4.1 to 5.8), or *p* value (<.001) for the Consultant *n*.

**Appendix 3. *Findings regarding variables other than emergency physician staffing***

As the only tertiary facility in the country, HGH has limited ability to transfer patients elsewhere. It is common for the system to have overcrowding, and the hospital tends to start each day relatively full before discharges are effected in the evening hours. This is the probable explanation for our finding that LOS was worst in the day shift (*i.e.* before any hospital discharges were made available to free up “back-end” capacity and offload the ED) and improved in later shifts.

The hospital system, including the ED, has been undergoing substantial construction over the past three years. While the ED operations effects from construction are not linear, the evaluation of study month as a covariate was judged to be a defensible surrogate for the gradually increasing construction effects (*e.g.* decreases in average number of functioning ED beds). This is the likely explanation for the fact that LOS was associated negatively with the passage of time.

One other time-related covariate, Friday status, was significant in the linear modeling and may require explanation. The study country is Muslim, and Friday is the holy day. At HGH, it has long been the case that ED operations are different on Fridays. The significant association of Friday status with LOS was not surprising and likely reflects the differing casemix as well as volume (since Friday status was significant even after adjusting for census).

Operational stress on the ED was assessed by looking at both census (which had predictably negative effects on LOS performance) and LWBS proportion. While the LWBS proportion could intuitively be seen as likely to improve LOS – these patients effectively decrease census and the study database reports their LOS as a missing value – it is the case at the study center that shifts with high LWBS tend to be shifts in which there are substantial operational pressures.

Patient factors contributing to LOS (at least in the linear model) included the proportions of low-acuity cases and Qatari nationals. As might be expected in a model that adjusts for overall census, higher proportions of low-acuity cases translate into a higher load of cases that are less likely to require time-consuming evaluation. With regard to proportion of Qatari nationals’ prediction of better (adjusted) LOS performance, the expatriates are less likely to have ready access to LOS-improving factors such as private admitting physicians or longitudinal care.
